# Vascular Risk Factors, Imaging, and Outcomes in Transient Ischemic Attack/Ischemic Stroke Patients with Neuroimaging Evidence of Probable/Possible Cerebral Amyloid Angiopathy

**DOI:** 10.1155/2021/9958851

**Published:** 2021-04-26

**Authors:** Qihui Zhang, Anxin Wang, Xia Meng, Xiaoling Liao, Yijun Zhang, Huiqing Hou, Lijuan Niu, Ruiping Li, Wenjuan Guo, Yongjun Wang

**Affiliations:** ^1^Department of Neurology, Beijing Tiantan Hospital, Capital Medical University, Beijing, China; ^2^China National Clinical Research Center for Neurological Diseases, Beijing, China; ^3^Department of Neurology, The Second Hospital of Hebei Medical University, Shijiazhuang, China; ^4^Beijing University of Chinese Medicine, Beijing, China

## Abstract

**Background:**

In TIA/ischemic stroke patients, the clinical significance of lobar microbleeds potentially indicating cerebral amyloid angiopathy (CAA) is unknown. We assessed vascular risk factors and outcomes, including cognition, in TIA/ischemic stroke patients with neuroimaging evidence of probable/possible CAA.

**Methods:**

This prospective cohort was conducted from August 2015 and January 2018 at 40 centers. 2625 participants were collected. Eligible participants were aged at least 55 years. Montreal Cognitive Assessment (MoCA) score is less than or equal to 26. A total of 1620 patients were included. 1604 (99.0%) and 1582 (97.7%) participants are followed up at 3 and 12 months. The primary outcomes were death or disability (mRS score, 3-6) and Montreal Cognitive Assessment (MoCA) at 3 months and 12 months. Demographic and vascular risk factors were measured at baseline (smoking, alcohol, diabetes, atrial fibrillation, hypertension, hypercholesterolemia, coronary artery disease, ischemic stroke, and transient ischemic attack). Blood samples were collected within 24 hours of admission. MRI was recommended for all patients. MoCA score was evaluated at baseline and follow-up.

**Results:**

In total, 291/1620 patients with ischemic stroke/TIA (32.7% female and mean age, 67.8 years) had neuroimaging evidence of probable/possible CAA. Higher age, history of hypertension, atrial fibrillation, ischemic stroke, alcohol, and high glucose at the admission were more common in the patients. Mean MoCA changed from 21.4 at 3 months (SD 5.2) to 22.3 at 12 months (SD 4.7), difference 0.3 (SD 3.8). At the 3-month and 12-month follow-up, there were significant differences in age, education level, and sex among different cognitive groups. Higher age, lower education (less than high school), and female sex were the predictors of changing in MoCA score from 3 months to 12 months. Moreover, age (more than 66 years) and education (less than high school) are strongly associated with MoCA at 3- and 12-month follow-up. 30 of 286 (10.5%) and 37 of 281 (13.2%) patients had poor outcome of death or disability (modified Rankin Scale score, 3-6) at follow-up 3 and 12 months. Cortical superficial siderosis (cSS) was associated with higher mRS at follow-up. cSS status, cSS count 1-2, cSS strictly lobar, and strictly deep might be the risks of outcomes in adjusted analyses.

**Conclusion:**

This study suggested that an increasing number of vascular risk factors and imaging markers were significantly associated with outcomes of TIA/ischemic stroke patients with CAA pattern. Male, young patients with high education should get better cognitive recovery.

## 1. Introduction

Cognitive impairment and degree of global disability after TIA or ischemic stroke is the most common outcome in some clinical trials. Cerebral amyloid angiopathy (CAA) is a small vessel disease of the brain, which leads to cognitive impairments in elderly people [1–3]. The impact of cerebral amyloid angiopathy to poststroke cognitive impairment and global disability in TIA/ischemic stroke patients is indeterminate.

Vascular risk factors, including smoking, hypertension, diabetes, hyperlipidemia, and atrial fibrillation (AF), are strongly associated with cognitive decline and global disability [4, 5]. Previous studies have comprehensively presented the influence risk of magnetic resonance imaging (MRI) to CAA. They focused on cerebral microbleeds (CMBs), white matter changes (WMHs), and cortical superficial siderosis (cSS) [6]. These three are often closely related. The consensus regarding neuroimaging marker for CAA is clinically meaningful [7]. The evaluation of the interactive effects of vascular risk and imaging markers is more important to the outcome of neuroimaging evidence of CAA pattern in ischemic intracerebral events (TIA and ischemic stroke).

In this study, we used data from China National Clinical Research Center for Neurological Diseases to mimic the design and to examine the China National Stroke Registry III Cognitive Subgroup (ICONS) participants at follow-up 3 and 12 months, when vascular risks and neuroimaging marker assessment appeared to be the most critical. We investigated cognitive impairment and the degree of global disability in patients with neuroimaging evidence of probable/possible CAA-related ischemic stroke or TIA. We hypothesized that vascular risk factors and imaging profile would be associated with the modified Rankin Scale (mRS) and that cognitive impairments at 3 and 12 months. On the basis of the highlighting the cognitive performance is associated with cerebral small-vessel disease at 12 months [8], we were interested in the factors predicting cognitive impairment in CAA pattern patients in TIA or ischemic stroke and comparing patients who improve between 3 and 12 months with those that do not.

## 2. Materials and Methods

### 2.1. Study Design

In this prospective study, we used data from the Impairment of Cognition and Sleep (ICONS) after acute ischemic stroke or transient ischemic attack study in Chinese patients conducted from August 2015 to January 2018. In 201 hospitals of the China National Stroke Registry III study, the steering committee of ICONS chose 40 hospitals nationwide to represent the population from each region of east, south, west, north, and center of Mainland China [9]. Consecutive IS or TIA in-hospital patients within 7 days after onset were enrolled in ICONS study. The study followed up 3 and 12 months.

### 2.2. Participants

In brief, the prospective cohort ICONS study recruited 2625 participants in China. The present analysis included 1620 selected patients, their age was ≥55 y, and MoCA score is ≤26. All patients tested brain MRI, including T2-weighted, T2^∗^-weighted gradient-recalled echo, and/or susceptibility-weighted imaging and fluid-attenuated inversion recovery sequences. 291 patients were clinically diagnosed CAA pattern by the Boston Criteria [10].

### 2.3. Data Collection

Demographic information and vascular risk factors, imaging, and cognitive tests were prospectively recorded at the time of study enrollment and then followed up for 3 months and 12 months. This has been described previously for the ICONS study.

### 2.4. Covariates

All 1620 visits include vascular risk factor assessments, defined atrial fibrillation, coronary artery disease, prior ischemic stroke, prior TIA, prior ICH, current smoking, and alcohol. The presence of diabetes (fasting glucose level ≥ 126 mg/dL, nonfasting glucose level ≥ 200 mg/dL, self-report of physician-diagnosed diabetes, or use of oral diabetes medications or insulin), hypertension (blood pressure was measured twice in resting state on different days, systolic blood pressure ≥ 140 mmHg and/or diastolic blood pressure ≥ 90 mmHg or physician-diagnosed hypertension and taking antihypertensive drugs), and hyperlipidemia [defined by patient having past history of hyperlipidemia, using a lipid-lowering agent including statins or patient diagnosed hyperlipidemia at admission as having serum cholesterol > 240 mg/dL or low − density lipoprotein cholesterol (LDL − cholesterol) levels ≥ 130 mg/dL] were recorded. For demographic factors, age, sex, and education level (college graduate or professional school, high school, junior school, primary school, and illiteracy) were self-reported. Body mass index (BMI) was calculated as weight in kilograms divided by height in meters squared. Plasma glucose, hemoglobin (Hgb), and platelet count (Plt) were measured. The modified Rankin Scale (mRS) is defined categorically with 7 levels of patient functional independence following a stroke, with scores ranging from 0 (fully independent) to 6 (death) [11]. According to the MoCA score, cognitive function was divided into three groups: normal (more than 26 score), mild (18-26 score), moderate, and severe (no more than 17 score) [12]. MoCA score was evaluated at admission and follow-up 3 and 12 months.

### 2.5. Imaging Acquisition and Analysis

Brain MRI scans, obtain as research studies at entry. All MRI images were reviewed centrally by two neuroradiologists.

MRI factors included diffusion-weighted imaging hyperintense lesions (DWIHLs), defined as regions of intraparenchymal hyperintensity on diffusion-weighted imaging, with associated hypointensity or matter hyperintensity (WMH) was evaluated visually on fluid-isointensity on apparent diffusion coefficients. White attenuated inversion recovery images using the Fazekas scale.

The number and location of cerebral microbleeds were identified according to the Brian Observer Micro Bleed Scale (BOMBS) on haem-sensitive MRI sequences [13], also known as T2^∗^ or gradient echo (GRE). There are 7 locations which should be assessed: contex/gray-white junction, subcortical white matter, basal ganglia grey matter, brainstem, cerebellum, internal/external capsule, and thalamus. The diagram demonstrates in the appropriate row for rating size (<5 mm, 5-10 mm). We followed the methods of Ashkan et al. [14]. The CMB count severity was coded a prior as absent (0 CMBs), mild (1-2 CMBs), moderate (3-10 CMBs), or severe (>10 CMBs) [15]. The CMB topography was coded as strictly lobar (with or without concurrent CMBs), strictly deep (deep and/or CMBs), or mixed (concurrent lobar and deep CMBs). The number and location of cSS were classified as CMBs.

### 2.6. Statistical Analysis

Patient demographic and MR imaging markers were compared between groups in cross-sectional analyses using a *χ*^2^ test or Fisher exact test for categorical variables and a *t*-test or Kruskal-Wallis test for continuous variables. We investigated the independent association between CAA pattern and outcomes (death or disability) with multivariable logistic regression analysis and adjusted for assigned treatment group, age, the presence of HTN, AF at baseline, and other covariates. These covariates were selected a prior based on the known predictors of these outcomes. The participants were followed up for 90 days and 12 months.

Separate univariable and multivariable ordinal regression analyses were used to assess the factors predicting of MoCA in patients, multivariable analysis adjusting for age, HTN, AF, prior ischemic stroke, alcohol, glucose, 3 m MoCA, and Fazekas scale.

All tests were 2 sided, and significance level was accepted at .05. All analyses were conducted using SAS version 9.4 (SAS Institute Inc., Cary, NC, USA).

### 2.7. Ethical Standards

The study received ethical approval by the ethics committee at Beijing Tiantan Hospital (No. KY2015-001-01). All patients or their legally authorized representative provided written informed consent.

## 3. Results

In August 2015-January 2018, 1620 of 2625 (61.71%) participants were enrolled in this study. Neuroimaging evidence of probable/possible CAA in TIA or ischemic stroke was present in 291 patients (32.7% female and mean [SD] age, 67.8).  Table 1 provides characteristics of the comparing patients in CAA pattern with non-CAA pattern patients. Among 20 variables in this analysis, 7 variables were differed significantly (age, history of hypertension, AF, ischemic stroke, alcohol, glucose, and MoCA score at 3 months).

Eighty-two percent (*n* = 241) of 291 CAA patients had CMBs. 76 patients had superficial siderosis (cSS). 26 patients had both CMBs and cSS.  Table 2 lists detailed neuroimaging findings in the CAA patients. Their median Fazekas scale score is 3.63 (IQR, 2-5). Compared to those without CAA, CMBs and cSS count in CAA patients were significant differences (*p* < 0.001 and *p* < 0.001). Among the CAA patients with no more than five CMBs, CMBs were located in 156 patients (53.6%) in the contex/grey-white junction, 47 patients (16.2%) in the subcortical white matter, 6 patients (2.1%) in the internal and external capsule, and 59 patients (20.3%) in the cerebellum. The number of cases with cSS located in the frontal, parietal, temporal, occipital, centrum semiovale, and cerebellum was 10 (3.4%), 14, (4.8%), 37 (12.7%), 13 (4.5%), 6 (2.1%), and 11 (3.8%).

Age was divided into two groups: 55-66 years and more than 66 years. Education was divided into three groups: more than high school (college graduate or professional school), high school, and less than high school (junior school, primary school, and illiteracy). Sex was divided into two groups: female and male. Follow-up for 3 months and 12 months showed that there were significant differences in age, education level, and sex among different cognitive groups (Table 3). In multivariable ordinal logistic regression analyses of the predictors of cognitive function changes, the MoCA score of CAA pattern patients with a follow-up of 3-12 months was associated with age (more than 66 years), education (less than high school), and female sex. In multivariable analyses of predictors of cognitive function at 12 months, the MoCA score at 12-month follow-up was related to age (more than 66 years) and education (less than high school) (Table 4).

During the follow-up of 3 months, 30 of 286 (10.5%) patients with CAA pattern had poor outcomes of death or disability (modified Rankin Scale score, 3-6), 24 of 244 (9.8%) patients with CAA pattern had CMBs, and 12 of 93 (12.9%) patients with CAA pattern had cSS. Patients with CMB count > 10 were increased at risk of death or disability (adjusted relative risk [aRR 2.17; 95% CI, 0.30-15.92]). In a multivariable analysis, patients with cSS were at increased risk of bad outcomes (adjusted relative risk [aRR 2.01; 95% CI, 0.85-4.77]), especially the cSS count 1-2 (adjusted relative risk [aRR 2.07; 95% CI, 0.88-4.90]), cSS topography including strictly lobar (adjusted relative risk [aRR 3.68; 95% CI, 1.22-11.17]), and strictly deep (adjusted relative risk [aRR 2.19; 95% CI, 0.59-17.35]) (Table 5).

37 of 281 (13.2%) patients with CAA pattern had poor outcomes of death or disability at follow-up 12 months. The lack of association between CMBs and the outcomes of patients with CAA pattern was consistent with the present. In multivariable analyses of the outcomes followed up 12 months, the various of cSS consistent at 3 months, cSS topography mixed (adjusted relative risk [aRR 1.25; 95% CI, 0.42-3.73]) were also associated with death or disability (Table 6).

## 4. Discussion

In this study, we have identified clinical and neuroimaging risk factor association with probable/possible CAA in TIA or ischemic stroke on admission. The increased risk with following characteristics: older age, history of hypertension, AF and ischemic stroke, alcohol, glucose on admission, and lower MoCA score at 3 months. More studies have been reported that the vascular factors such as age and history of hypertension are associated with of CAA [5, 6, 16]. Our findings suggested that we should pay more attention to the history of ischemic stroke and others. Among the non-CAA patients, the proportion with hypertension was surprisingly low (62.7%). This may be explained that the non-CAA group with nonhypertensive ICHs is due to vascular malformations.

In the multivariate analyses, age is a risk factor associated with CAA pattern. In addition, age is also a strong predictor factor of development of cognitive impairment in the first 1 year after diagnosis of CAA pattern in TIA/ischemic stroke. The increase with age is well known as the prevalence of cognitive impairment in Alzheimer's disease and other neurodegenerative diseases [17, 18]. The MoCA score would be higher in patients who improve between 3 and 12 months with lower age, female sex, and higher education. It might be interesting that they did contribute to prediction of cognitive decline would be recovering in patients with CAA pattern. Though sex may not be a strong predictive factor with MoCA at 12-month follow-up, but the female sex patients may still recover better at 3 months and have higher scores at 12 months. With higher level education, patients should get more better managements, more preventive interventions, and appropriate treatment to improve cognitive performance, as previously been reported in fewer years [19].

In this cohort study, CAA pattern was associated with the presence of WMHs. We observed greater death or disability at 3 and 12 months in the patients with cSS, compared with those with CMBs, except CMB counts > 10. It seems that a strong predictor of outcomes of CAA pattern was associated with cSS count and topography, irrespective of whether the outcomes at 3 months or 1 year. Some studies observed that cSS affected both the supra- and the infratentorial compartments associated with CAA patients [20].

### 4.1. Strengths and Limitations

Our study has some limitations. Though we analyzed the imaging markers including WMH, CMBs, and cSS, we did not collect other neuroimaging various assessments for the outcomes of death or disability at 3 and 12 months. The cSS sample size was smaller than expected to get more information. It seems to limit us to correctly assess factors though cSS count and topography. Moreover, this study only included a follow-up of the first one year after diagnosis, and few patients with cognitive clinical symptoms developed or recovered slowly. Our findings should therefore be considered in the context of predicting cognitive impairment with longer follow-up. We should further validate the factors and the predicting risks in other cohorts and explain further in the appropriate clinical context.

## 5. Conclusions

In this study, age, history of hypertension, and ischemic stroke were strong factors associated with CAA pattern, whereas higher educational level, female sex, and young age maybe recover the cognitive impairment. cSS was significant for outcomes. These findings may provide some support of the outcomes of the CAA pattern in TIA/ischemic stroke.

## Figures and Tables

**Table 1:** Characteristics of TIA/ischemic stroke patients with neuroimaging evidence of probable/possible CAA pattern.

Characteristic	All patients (*n* = 1620)	CAA pattern (*n* = 291)	Non-CAA pattern (*n* = 1329)	*p* value
Age, mean (SD), y	66.5 (7.6)	67.8 (7.7)	66.2 (7.5)	0.001
Sex (female), No., %	490 (30.3%)	95 (32.7%)	395 (29.7)	0.33
BMI, mean (SD)	24.8 (3.2)	24.9 (3.2)	24.8 (3.2)	0.52
HTN No., %	1045 (64.5%)	212 (72.9%)	833 (62.7%)	0.001
DM No., %	387 (23.9%)	65 (22.3%)	322 (24.2%)	0.49
AF No., %	111 (6.9%)	29 (10.0%)	82 (6.2%)	0.02
Hyperlipidemia No., %	148 (9.1%)	23 (7.9%)	125 (9.4%)	0.42
Coronary artery disease No., %	11 (0.7%)	4 (1.4%)	7 (0.5%)	0.11
Prior ischemic stroke No., %	391 (24.1%)	91 (31.3%)	300 (22.6%)	0.002
Prior TIA No., %	47 (2.9%)	10 (3.4%)	37 (2.8%)	0.83
Current smoking No., %	505 (31.2%)	86 (29.6%)	419 (31.5%)	0.51
Alcohol No., %	239 (14.8%)	32 (11.0%)	207 (15.6%)	0.046
Education level No., %				0.56
>high school	118 (7.3%)	22 (7.6%)	96 (7.2%)	
High school	344 (21.2%)	55 (18.9%)	289 (21.8%)	
<high school	1158 (71.5%)	241 (73.5%)	944 (71.0%)	
Initial mRS, mean (SD)	1.8 (1.3)	1.8 (1.3)	1.8 (1.3)	0.44
Initial MoCA, mean (SD)	19.2 (5.3)	18.9 (5.4)	19.3 (5.3)	0.24
Glucose mean (IQR), mg/dL^b^	6.3 (2.4)	6.1 (2.4)	6.4 (2.4)	0.03
Hgb g/L ^c^	140.6 (16.2)	141.1 (16.6)	140.5 (16.1)	0.50
Plt ∗10^9^/L^d^	206.0 (56.8)	199.0 (58.7)	207.5 (56.3)	0.06
3 m MoCA^e^	22.1 (5.0)	21.4 (5.2)	22.3 (4.9)	0.03
12 m MoCA^f^	22.4 (4.9)	22.3 (4.7)	22.4 (5.0)	0.85

Abbreviations: CAA: cerebral amyloid angiopathy; BMI: body mass index; HTN: hypertension; DM: diabetes; AF: atrial fibrillation; TIA: transient ischemic attack; mRS: modified Rankin Scale; MoCA: Montreal Cognitive Assessment; Hgb: hemoglobin; Plt: platelet count. ^b^Data were missing for 227 patients. ^c^Data were missing for 9 patients. ^d^Data were missing for 12 patients. ^e^Data were missing for 233 patients. ^f^Data were missing for 724 patients.

**Table 2:** MR imaging findings by status of CAA pattern in TIA/ischemic stroke patients.

Variable	CAA pattern (*n* = 291)	Without CAA pattern (*n* = 1329)
Fazekas scale score, mean (IQR)^a^	3.63 (2-5)	2.61 (1-4)
CMB count (No., %)	241 (82.3%)	38 (2.9%)
CMB topography (No., %)		
Contex/grey-white junction		
Number < 5 mm	156 (53.6%)	1 (0.1%)
Number 5-10 mm	14 (4.8%)	0 (0)
Subcortical white matter		
Number < 5 mm	47 (16.2%)	1 (0.1%)
Number 5-10 mm	2 (0.7%)	0 (0)
Internal and external capsule		
Number < 5 mm	6 (2.1%)	12 (0.9%)
Number 5-10 mm	0 (0)	0 (0)
Cerebellum		
Number < 5 mm	59 (20.3%)	0 (0)
Number 5-10 mm	5 (1.7%)	0 (0)
cSS count (No., %)	95 (32.7%)	42 (3.2%)
cSS topography (No., %)		
Frontal	10 (3.4%)	1 (0.1%)
Parietal	14 (4.8%)	1 (0.1%)
Temporal	37 (12.7%)	2 (0.2%)
Occipital	13 (4.5%)	0 (0)
Centrum semiovale	6 (2.1%)	0 (0)
Cerebellum	11 (3.8%)	0 (0)

Abbreviations: CMB: cerebral microbleed; cSS: cortical superficial siderosis. ^a^Data were missing for 193 patients.

**Table 3:** Changes of MoCA score in TIA/ischemic stroke patients with CAA pattern at 3 and 12 months.

3 months (*n* = 1380)					12 months (*n* = 896)				
Variable	MoCA (>26)	MoCA (18-26)	MoCA (≤17)	*p* value	Variable	MoCA (>26)	MoCA (18-26)	MoCA (≤17)	*p* value
Age				<0.001	Age				<0.001
55-66 y (No., %)	168 (64.1%)	509 (56.8%)	100 (43.7%)		55-66 y (No., %)	113 (64.6%)	356 (60.3%)	54 (41.2%)	
>66 y (No., %)	94 (35.9%)	387 (43.2%)	129 (56.3%)		>66 y (No., %)	62 (35.4%)	234 (39.7%)	77 (58.8%)	
Education				<0.001	Education				<0.001
>high school (No., %)	22 (8.4%)	70 (7.8%)	3 (1.3%)		>high school (No., %)	19 (10.9%)	47 (8.0%)	3 (2.3%)	
High school (No., %)	67 (25.6%)	196 (21.9%)	31 (13.5%)		High school (No., %)	42 (24.0%)	122 (20.7%)	19 (14.5%)	
<high school (No., %)	173 (66.0%)	630 (70.3%)	195 (85.2%)		<high school (No., %)	114 (65.1%)	421 (71.4%)	109 (83.2%)	
Sex				<0.001	Sex				<0.001
Male (No., %)	190 (72.5%)	649 (72.4%)	132 (57.6%)		Male (No., %)	129 (73.7%)	423 (71.7%)	78 (59.5%)	
Female (No., %)	72 (27.5%)	247 (27.6%)	97 (42.4%)		Female (No., %)	46 (26.3%)	167 (28.3%)	53 (40.5%)	

**Table 4:** Factors predicting of MoCA score improvement in TIA/ischemic stroke patients with CAA pattern at 3 and 12 months.

3 months			12 months		
Variable	OR (95% CI)	*p* value	Variable	OR (95% CI)	*p* value
Age (>66 y)	1.51 (1.19-1.92)	0.001	Age (>66 y)	1.66 (1.23-2.24)	0.001
Education (high school)	0.70 (0.52-0.93)	0.02	Education (high school)	0.80 (0.56-1.16)	0.24
Education (>high school)	0.52 (0.32-0.82)	0.005	Education (>high school)	0.45 (0.26-0.77)	0.004
Sex (female)	1.43 (1.11-1.85)	0.006			

Education (>high school): college graduate or professional school.

**Table 5:** Outcomes of CAA pattern in TIA/ischemic stroke patients at 3 months.

Outcomes	Death or disability (mRS 3-6)		
	No. of events	Crude RR (95% CI)	Adjusted RR (95% CI)
CMB status^a^			
No CMB (*n* = 42)	6/42	1 (reference)	1 (reference)
CMB (*n* = 244)	24/244	0.66 (0.25-1.71)	0.33 (0.11-0.98)
CMB count^a^			
None (*n* = 42)	6/42	1 (reference)	1 (reference)
1-2 (*n* = 165)	13/165	0.51 (0.18-1.44)	0.27 (0.08-0.86)
3-10 (*n* = 72)	9/72	0.86 (0.28-2.60)	0.36 (0.10-1.31)
>10 (*n* = 7)	2/7	2.40 (0.38-15.33)	2.17 (0.30-15.92)
CMB topography^a^			
None (*n* = 67)	8/67	1 (reference)	1 (reference)
Strictly lobar (*n* = 133)	11/133	0.67 (0.25-1.74)	0.50 (0.17-1.46)
Strictly deep (*n* = 10)	1/10	0.82 (0.09-7.35)	0.57 (0.06-5.73)
Mixed (*n* = 76)	10/76	1.12 (0.41-3.02)	0.70 (0.22-2.18)
cSS status^b^			
No cSS (*n* = 192)	18/192	1 (reference)	1 (reference)
cSS (*n* = 93)	12/93	1.43 (0.66-3.11)	2.01 (0.85-4.77)
cSS count^b^			
None (*n* = 192)	18/192	1 (reference)	1 (reference)
1-2 (*n* = 89)	12/89	1.51 (0.69-3.28)	2.07 (0.88-4.90)
3-10 (*n* = 4)	0/4	—	—
>10 (*n* = 0)	0	0	0
cSS topography^b^			
None (*n* = 192)	18/192	1 (reference)	1 (reference)
Strictly lobar (*n* = 35)	6/35	2.00 (0.73-5.46)	3.68 (1.22-11.17)
Strictly deep (*n* = 10)	2/10	2.42 (0.48-12.26)	2.19 (0.59-17.35)
Mixed (*n* = 48)	4/48	0.88 (0.28-2.73)	0.92 (0.25-3.47)

Abbreviations: RR: relative risk; CMB: cerebral microbleed; cSS: cortical superficial siderosis. Multivariable analysis adjusted for age, HTN (history of hypertension), AF (history of atrial fibrillation), prior ischemic stroke, alcohol, glucose, 3 m MoCA, and Fazekas scale. ^a^Data were missing for 5 patients. ^b^Data were missing for 6 patients.

**Table 6:** Outcomes of CAA pattern in TIA/ischemic stroke patients at 12 months.

Outcomes	Death or disability (mRS 3-6)		
	No. of events	Crude RR (95% CI)	Adjusted RR (95% CI)
CMB status^a^			
No CMB (*n* = 42)	8/42	1 (reference)	1 (reference)
CMB (*n* = 239)	29/239	0.59 (0.25-1.39)	0.26 (0.09-0.71)
CMB count^a^			
None (*n* = 42)	8/42	1 (reference)	1 (reference)
1-2 (*n* = 162)	16/162	0.47 (0.18-1.18)	0.22 (0.08-0.65)
3-10 (*n* = 70)	12/70	0.88 (0.33-2.37)	0.34 (0.10-1.10)
>10 (*n* = 7)	1/7	0.71 (0.07-6.74)	0.46 (0.04-4.76)
CMB topography^a^			
None (*n* = 68)	11/68	1 (reference)	1 (reference)
Strictly lobar (*n* = 128)	12/128	0.54 (0.22-1.29)	0.36 (0.14-0.96)
Strictly deep (*n* = 10)	2/10	1.30 (0.24-6.94)	0.94 (0.15-5.75)
Mixed (*n* = 75)	12/75	0.99 (0.40-2.41)	0.54 (0.19-1.53)
cSS status^b^			
No cSS (*n* = 188)	24/188	1 (reference)	1 (reference)
cSS (*n* = 92)	13/92	1.12 (0.54-2.33)	1.63 (0.73-3.67)
cSS count^b^			
None (*n* = 188)	24/188	1 (reference)	1 (reference)
1-2 (*n* = 89)	13/89	1.17 (0.57-2.42)	1.66 (0.74-3.73)
3-10 (*n* = 3)	0/3	—	—
>10 (*n* = 0)	0	0	0
cSS topography^b^			
None (*n* = 188)	24/188	1 (reference)	1 (reference)
Strictly lobar (*n* = 35)	5/35	1.14 (0.40-3.22)	2.05 (0.67-6.29)
Strictly deep (*n* = 10)	2/10	1.71 (0.34-8.53)	2.09 (0.39-11.18)
Mixed (*n* = 47)	6/47	1.00 (0.38-2.61)	1.25 (0.42-3.73)

Abbreviations: RR: relative risk; CMB: cerebral microbleed; cSS: cortical superficial siderosis. Multivariable analysis adjusted for age, HTN (history of hypertension), AF (history of atrial fibrillation), prior ischemic stroke, alcohol, glucose, 3 m MoCA, and Fazekas scale. ^a^Data were missing for 10 patients. ^b^Data were missing for 11 patients.

## Data Availability

In this study, we used data from China National Clinical Research Center for Neurological Diseases to mimic the design and to examine the China National Stroke Registry III Cognitive Subgroup (ICONS). The data is available on request to China National Clinical Research Center for Neurological Diseases.
